# Investigating employee perceptions: Association between recognized individual talents and social wellbeing

**DOI:** 10.3389/fpsyg.2022.959559

**Published:** 2022-07-25

**Authors:** Janina M. Björk, Pernilla Bolander, Anna K. Forsman

**Affiliations:** ^1^Department of Developmental Psychology, Faculty of Education and Welfare Studies, Åbo Akademi University, Vaasa, Finland; ^2^Department of Management and Organization, Stockholm School of Economics, Stockholm, Sweden; ^3^Department of Health Sciences, Faculty of Education and Welfare Studies, Åbo Akademi University, Vaasa, Finland

**Keywords:** employee social wellbeing, employee welfare, positive psychology, inclusive talent management, individual talents, manufacturing industry, cross-sectional study, integrative perspective

## Abstract

**Background:**

Organizations worldwide increasingly adopt inclusive talent management, and this approach appears to rhyme particularly well with the Nordic welfare model. Questions about its value remain understudied, however. The inclusive approach is rooted in positive psychology and focuses on recognizing each employee's individual talents and assessing whether they fit the long-term needs of the organization, since a fit is assumed to be associated with employees' wellbeing. In the present study, we test this assumption focusing specifically on a key talent management practice, talent identification, and the social dimension of employee wellbeing.

**Method:**

Data were collected through an employee survey conducted within the Finnish units of four international manufacturing organizations and analyzed using logistic regression (*n* = 618).

**Results:**

We found that the recognition of individual talents for long-term deployment by the organization is positively associated with social wellbeing in terms of supervisor support and social climate in the work unit, as perceived by the employees.

**Conclusion:**

Our results tentatively suggest that inclusive talent management creates value through the identification of employees' individual talents as this practice can be associated with their enhanced wellbeing.

## Introduction

During the past two decades, talent management (TM) has become an increasingly common phenomenon in working life. Organizations worldwide continue to make significant investments in TM yet questions about its value remain understudied (Sparrow and Makram, 2015; Claus, 2019; King and Vaiman, 2019).

There are two fundamentally different approaches to TM. Currently most common in both research and practice, the exclusive approach defines talent as rare, emphasizing that practices should be targeted toward a small, elite subset of the organization's employees (Gallardo-Gallardo et al., 2013; Makarem et al., 2019). This approach advocates control and quantification of employees' normative performance (Boudreau and Ramstad, 2005) and disproportionate conferment of organizational resources (e.g., development opportunities, job rotations, high status) to the designated talents. In turn, the selected few are above all else expected to generate disproportionate economic value, for example by displaying favorable attitudes (e.g., high organizational commitment) and behaviors (e.g., high work effort; Gelens et al., 2014) toward the organization. Building on social exchange theory, a growing body of research is testing this assumption. Although TM encompasses a broad range of practices, including identifying, developing, and retaining talents (Bolander et al., 2017), much of the research has focused on one specific practice, namely talent identification, and associated outcomes at the level of the individual employee. So far, mixed effects have been reported, evidencing both positive and negative reactions in employees that have been identified as talents and both significant and non-significant differences in reactions between talents and non-talents (De Boeck et al., 2018).

In contrast, the inclusive approach recognizes ‘the full range of talent in the organization' (Swailes et al., 2014, p. 534) and ‘aims at investing in a broad variety of different talents' (Meyers, 2016, p. 4). Leveraging the literature on positive psychology (Seligman and Csikszentmihalyi, 2000), a scientific discipline that is devoted to what is good and well-functioning in human life, the inclusive approach defines talent as individual strengths or personal characteristics which allow individuals to be at their personal best (Peterson and Seligman, 2004; Wood et al., 2011; Quinlan et al., 2012). That is, it advocates that talent should be regarded from a within- and not a between-person perspective. Moreover, it stresses that organizations should strive to find and offer positions to all employees that give the best opportunity for them to use their individual talents (Swailes et al., 2014; Meyers, 2016). Focusing on the employees' individual talents, the inclusive approach is assumed to create multiple values in today's dynamic working life, such as employee wellbeing, quick learning processes, and high productivity (Peterson and Seligman, 2004; Swailes et al., 2014; Meyers et al., 2020). Compared to the empirical literature examining the value of exclusive TM, that on inclusive TM is scant (Gallardo-Gallardo and Thunnissen, 2016). This is problematic, since various contextual factors are driving a growing number of organizations to adopt more inclusive TM practices (Meyers, 2016; Gallardo-Gallardo et al., 2020; Meyers et al., 2020).

The current study is based on survey data from four organizations with headquarters in Finland, all of them adopting an inclusive approach to TM. We focused on a central TM practice (talent identification) and how this practice is associated with a key dimension of employee wellbeing (social wellbeing), the single most important value of the inclusive approach (Swailes et al., 2014). Specifically, with the study presented here, we seek to bring light to the potential value of recognizing employees' individual talents for their social wellbeing, that is, the experienced quality of interpersonal relationships, as well as perceived trust and social support at work (Grant et al., 2007; Van De Voorde et al., 2012; Guest, 2017).

### Theoretical framework

#### Promotion of an inclusive understanding of talent management in contemporary Nordic working life

The Nordic countries share several societal values and security systems alike that distinguish them from other countries in Europe and across the globe. Well-developed public sectors provide extensive welfare services, for example in terms of healthcare, education, and childcare. According to the research tradition initiated by Three Worlds of Welfare Capitalism (Esping-Andersen, 1990), there are three different types of regimes; the Social Democratic, the Conservative, and the Liberal regime type. The first type dominates in the Nordic context, and it is characterized by a comprehensive system of social protection with generous benefits based on citizenship (right-based Universalism) and upholding public provisions of services. For example, a unique future of the Nordic welfare system is the family policy, which includes paid parental leave, public, subsidized day care, and free education. This family policy has resulted in a high proportion of dual-earner couples and a highly educated workforce. The Nordic welfare system has strongly contributed to longstanding working life traditions of inclusive, egalitarian, and participative management (Ahl et al., 2018). For example, “co-workership” is a salient concept across Nordic organizations and refers to high degrees of employee responsibility, engagement, participation, and influence. In other words, “co-workership” emphasizes the value employees bring to the organization (Kilhammar and Ellström, 2015). The Nordic welfare system is also reflected in organizational leadership in terms of an emphasis on health promotion (Eriksson, 2011).

It has been argued that organizations' HR practices, including TM practices, are embedded in, and formed by, the national and regional context in which they operate (Gallardo-Gallardo et al., 2020). Indeed, potential effects of different TM practices are now being discussed in relation to various national and regional contexts (cf. Iles et al., 2010; Stahl et al., 2012). Against the backdrop described above, cultural traditions and values in the Finnish work context can present serious challenges to both the implementation and the effectiveness of exclusive TM practices (Sumelius et al., 2020). Inclusive TM, on the other hand, is primarily motivated by the welfare of employees and by extension society at large (Swailes et al., 2014), and it is therefore likely to better fit the Finnish work context.

According to the Global Talent Competitiveness Index (GTCI) 2020. Finnish organizations to a great extent implement practices associated with inclusive TM, such as enabling, growing, and retaining talent, and Finland is one of the leading countries when it comes to matching the skills of people with the needs of the economy (Lanvin and Monteiro, 2020). Current trends on the labor market reinforce this focus on inclusive TM practices. For example, the Finnish workforce is increasingly age diverse (Pahkin et al., 2008), which creates a need for TM activities that target workers of various ages. Another challenge lies in a general skill shortage. With rapid technological advances, knowledge becomes quickly outdated and many sectors therefore face a shortage of well-educated workers (Rauhut and Kahila, 2008; Niemi et al., 2021). When there is a talent shortage in the labor market, it is difficult to attract new competent members to the organization's workforce (Ahl et al., 2018). This is an especially pressing issue in the Finnish work context, which is dominated by knowledge-intensive industries and services (Sumelius et al., 2020; Niemi et al., 2021). In fact, Finland scores significantly lower on the ability to attract talent, especially from abroad, than it does on any other parameter in the GTCI 2020 (Lanvin and Monteiro, 2020). In this situation, international research shows that the best available option for many organizations is to put into place practices that broadly aim to retain and develop their current workforce, while also identifying organization-specific turnover drivers (Allen et al., 2010). In addition to the talent shortage that many organizations face, in Finland and in other countries around the world, there are increased expectations of social responsibility on organizations. It is therefore of strategic importance for organizations to include all types of people and to present them with equal opportunities (Ahl et al., 2018). In sum, contemporary Nordic working life promotes an inclusive understanding of TM, which encourages organizations operating in this context to adopt an inclusive TM approach.

#### Creating value through talent identification in inclusive talent management

According to King (2016), talent identification is a central practice to which employees respond as it signals organizational priorities for talent in the organization. That is, talent identification may be interpreted by the employees as containing important information regarding their individual standing within a particular organizational context, and they will respond according to their interpretations.

Scholars who represent the dominant, exclusive approach to TM commonly operationalize talent identification by normative talent status to distinguish exceptional key employees from the rest (De Boeck et al., 2018). In their examination, they usually rely on information from the organization regarding the employee's assigned talent status, although a few studies exist that have investigated talent status as perceived by the individual employee (e.g., Björkman et al., 2013). Further, these scholars are interested in associations between talent status and employee attitudes that can be considered important for the performance of the organization, such as employee commitment, satisfaction, and motivation (Dries and Pepermans, 2007; Gelens et al., 2014; Seopa et al., 2015).

Conversely, scholars who represent the critical, inclusive approach to TM regard talent identification to be about recognizing each employee's individual talents in relation to a particular organizational context (Swailes et al., 2014; Meyers, 2016). To understand how talent identification works from an inclusive approach, the literature on person-environment fit can be helpful (e.g., Kristof-Brown et al., 2005). Two central types of fit discussed in this literature are when an employee's individual characteristics are compatible with the characteristics of the organization that it works in (person-organization fit; P-O fit) and with the tasks that it performs (person-job fit, P-J fit). Empirical research has demonstrated associations between both types of fit and positive employee attitudes and behaviors (e.g., Kristof-Brown et al., 2005), including wellbeing (Roczniewska et al., 2018).

In the context of TM, individuals who fit into the organization and the job are expected to be at their personal best. Rooted in the basic propositions of positive psychology, inclusive TM regards “personal best” to entail superior performance and wellbeing from a within-person perspective, meaning that it stands in comparison with the individual's own general performance and wellbeing (Swailes et al., 2014; Meyers, 2016).

Therefore, a vital part of inclusive TM is to fit employees into jobs that match their individual talents. Being identified as talent by an organization that has adopted inclusive TM thus entails possessing characteristics and abilities that the organization needs for long-term success. In turn, not being identified as talent by the organization simply means that the employee is currently not provided with sufficient training and opportunity or that the employee could find a better fit in another organization to fully realize its potential. Although not empirically tested, a likely employee response to inclusive talent identification “is one of enhanced happiness, fulfillment and wellbeing among other positive affective states” (Swailes et al., 2014, p. 535). In sum, talent identification in inclusive TM is focused on identifying employees' individual talents and how these are compatible with the organization and the job, as a fit likely enhances employee wellbeing and performance alike (Meyers, 2016).

#### The present study: Differences in perceived social wellbeing among self-reported talents and non-talents

The aim of this study is to explore the association between employees' perception of whether they have been recognized by the organization for their individual talents and their perceived social wellbeing at work. In this examination, we operate from a within-person perspective on talent rather than a between-person perspective, as this study is undertaken in organizations that have adopted the inclusive TM approach. Specifically, we refer to employees who perceive that their individual talents have been identified for long-term deployment by the organization as self-reported talents, and we refer to those who perceive that their individual talents have not been identified or do not know whether they have been identified as self-reported non-talents. The employee perspective was utilized in this examination since we adhere to the growing number of scholars who argue that the effect of TM does not stem from the actual practices but from the perceptions individual employees have of those practices (e.g., Thunnissen, 2016).

We chose to focus on employees' social wellbeing as this is increasingly considered to be a key dimension of employee wellbeing (Grant et al., 2007). For example, modern conceptual models of employee wellbeing incorporate social functioning (e.g., Appelbaum et al., 2000; Purcell and Kinnie, 2007; Boxall and Purcell, 2008). Building on Grant et al. (2007), Van De Voorde et al. (2012), and Guest (2017), we define social wellbeing at work as the employees' experienced quality of their interpersonal relationships, as well as perceived trust and social support provided in the work community that they are part of. Like all dimensions of employee wellbeing, social wellbeing is considered of high value in organizations that have adopted an inclusive TM approach, as this approach is primarily motivated by employees' welfare (e.g., Swailes et al., 2014).

Specifically, we examine the employees' perception of whether they have been recognized for their individual talents in relation to two aspects of their perceived social wellbeing at work. The first aspect, perceived supervisor support, refers to the perception that the nearest superior supports and helps the employees when needed, listens to the employees when they are experiencing work-related problems, and appreciates the employees' work achievements. The second aspect is perceived social climate in the employees' work unit. A positive social climate is characterized by a supportive, trustful work atmosphere in the employees' immediate work environment in which they can feel relaxed and comfortable. Thus, while the first aspect clearly states who gives support to whom, the second denotes reciprocity and a general measure of the social climate in the work unit (Sundin et al., 2006). Note that by adopting these two measures of social wellbeing at work in the analysis, we incorporate two distinct aspects of social wellbeing (Van De Voorde et al., 2012): employees' interactions and relationships with supervisors (who act as agents of the organization; Rhoades and Eisenberger, 2002) and with other employees. Also, we control for three demographic background variables in the analysis: age, gender, and supervisory position.

Drawing on the literature on talent identification in inclusive TM, which leverages the basic propositions of positive psychology as well as P-O and P-J fit theory, we formulated the following hypotheses to test one of the most frequent assumptions made in inclusive TM: that inclusive TM creates value through the association between the organization's recognition of employees' individual talents and enhanced employee (social) wellbeing:

*H1*: Self-reported talent is positively associated with employee social wellbeing, in terms of supervisor support.
*H2*: Self-reported talent is positively associated with employee social wellbeing, in terms of social climate in the work unit.

## Materials and methods

### Study context

The current study was situated in the bilingual Finnish region of Ostrobothnia. It is known for its high export rate and internationality is emphasized. The unemployment rate of Ostrobothnia is the lowest of all regions in Finland, and a significant number of open positions are not filled due to talent shortage [ELY (Centre for Economic Development, 2021; Niemi et al., 2021]. In addition, as described in the introduction, traditional values of employee welfare are highly held all around Finland and the other Nordic countries (Ahl et al., 2018). These factors strongly contribute to an inclusive understanding of talent identification, development, and retention among companies in Ostrobothnia.

### Participants and procedure

The data that form the basis for this study were collected in June 2018 in four international manufacturing organizations with headquarters based in Finland. The organizations represented the food production industry, mechanical and industrial engineering, as well as the automotive industry.

We distributed the invitation and the web-based double-blinded survey to the employees with the help of the HR managers of the participating companies. This survey design guaranteed respondent anonymity. In Finland, the Medical Research Act and Decree (488/1999) regulates medical research involving human beings. For non-medical research involving human participants, the Finnish National Board on Research Integrity TENK has issued a set of guidelines on the ethical principles [The Finnish National Board on Research Integrity (TENK), 2019], which were strictly followed in the design and execution of this study. Further, we customized the questionnaire for each company and distributed it in three language versions (Finnish, Swedish, and English). This design enabled employees representing different language groups to anonymously participate in the study and allowed the inclusion of some company-specific terminology, such as the company unit names. The total number of distributed questionnaires were 1,520. We received 618 responses, resulting in a response rate of 41%.

With regards to the distribution of socio-demographic characteristics among the study sample, respondents aged 30 or under were 126 (20.4%), respondents aged 31–40 were 188 (30.4%), respondents aged 41–50 were 173 (28%), and respondents older than 50 were 131 (21.2%). The sample consisted of 402 male (65%) and 216 female (35%) respondents. The number of respondents reporting that they are in a supervisory position was 164 (26.5%), compared to 454 respondents reporting that they are not (73.5%).

With regards to the studied independent variable (i.e., self-reported talent), 351 respondents (56.8%) experienced that they are identified as talent by the organization, and 267 that they are not or that they don't know (43.2%). In general, respondents reported high levels of social wellbeing (operationalized by our two dependent variables), as the mean score for the scale measuring supervisor support was 3.72 (standard deviation: 1.00), and for the scale measuring social climate in the own work unit was 3.68 (standard deviation: 0.84).

There were no internal missing values for the variables included in the analyses since the web-based questionnaire used in the data collection could only be submitted by the respondents if they had selected one of the existing response options to every question.

### Measures

#### Dependent variables

Social wellbeing was operationalized by perceived social support from supervisors and by perceived social climate in the own work unit. In line with previous research conducted in the Nordic work context (e.g., De Cuyper et al., 2011; Finne et al., 2014; Jönsson et al., 2015; Sigursteinsdottir et al., 2020), two different instruments from the QPSNordic-questionnaire (Lindström et al., 2000) were used in this study to measure social support from supervisors and social climate. This questionnaire was developed for investigating psychological and social factors at work, and adequate levels of internal consistency and test-retest reliability have been reported for both instruments (Dallner et al., 2000). We carefully chose these instruments from the QPSNordic as they are well suited for the Nordic context.

Both perceived supervisor support and perceived social climate in the own work unit were measured using three item-instruments. Sample items from these instruments are: “If needed, can you get support and help with your work from your nearest superior?,” and “Is the climate in your work unit encouraging and supportive?”. Answers ranged from 1 (“Very seldom or never” or “Very little or not at all”) to 5 (“Very often or always” or “Very much”).

Cronbach's α within our sample was 0.87 and 0.81 for the respective instruments, which indicated good reliability (Field, 2013). The instruments consist of the mean of the response to the three respective items (i.e., the sum of the response divided with the number of items), thus ranging from 1 to 5.

#### Independent variable

To capture the employees' perception of whether they have been recognized for their individual talents by the organization, we first provided them with a definition of talent and clarified that their potential identification as talent could be communicated formally or informally. The definition was: “Talents are employees perceived to possess characteristics and abilities that the organization needs for long-term success. The identification can be formal (e.g., through Your organization's HR processes) or informal (e.g., through Your manager talking to You).” We then asked the respondents to answer the following question: “Do you experience that you are identified as talent by your organization?”. Based on the respondents' answers to this question, we created a variable with two categories, in which respondents who answered “Yes” were coded as 1, and “Don't know or No” were coded as 0.

#### Control variables

We included three socio-demographic individual-level variables. These were gender (1 = man, 0 = woman), chronological age (1 = 30 years or under, 2 = 31–40 years, 3 = 41–50 years, 4 = over 50 years), and supervisory position (1 = yes, 0 = no).

### Statistical analyses

First, preliminary descriptive and correlation analyses (see Table 1) were conducted. The analyses showed that the correlations (*p* < 0.01; *p* < 0.001) in the model were below 0.70. This indicated no signs of significant collinearity problems, which correlations above 0.80 tend to indicate (Field, 2013).

**Table 1:** Descriptive statistics and correlations among study variables (*N* = 618).

	**M**	**SD**	**1**	**2**	**3**	**4**	**5**	**6**
1. Perceived supervisor support^a^	3.72	1.00	–					
2. Perceived social climate^a^	3.68	0.84	0.55***	–				
3. Age^b^	2.5	1.04	0.00	0.01	–			
4. Gender^c^	0.65	0.48	0.02	0.07	0.03	–		
5. Supervisory position^c^	0.27	0.44	0.06	0.04	0.19***	0.12**	–	
6. Self-reported talent^c^	0.57	0.50	0.28***	0.24***	0.17***	0.07	0.24***	–

^**^p < 0.01;

^***^p < 0.001.

^a^Mean scores for the scales measuring perceived supervisor support and perceived social climate (ranging from 1 to 5, where 1 indicates low social wellbeing and 5 high social wellbeing).

^b^Age is a categorical variable (1 = 30 years or under, 2 = 31–40 years, 3 = 41–50 years, 4 = over 50 years).

^c^Gender (female = 0, male = 1), supervisory position (no = 0, yes = 1), and self-reported talent (talent = 1, non-talent = 0) are dichotomous variables.

Second, we conducted binary, stepwise logistic regression analysis (see e.g., Field, 2013; Brace et al., 2016; Harris, 2020) to test the study hypotheses. Perceived supervisor support was the dependent variable in the first regression model, and perceived social climate in the second model. Before conducting the logistic regression analysis, we dichotomized the social wellbeing variables based on the mean scores of the respondents. Those scoring above 4 were categorized into the high supervisor support group (coded as 1), and those scoring 4 or below this were categorized into the low or medium supervisor support group (coded as 0). Similarly, the respondents were categorized into the positive social climate group = 1, and into the negative or neutral social climate group = 0. The control variables (age, gender, and supervisory position) as well as the independent variable (self-reported talent) were not further recoded for this step of the analysis.

In line with our study aim, we were particularly interested in predicting whether a self-reported talent belongs to the category in which respondents perceive high levels of social wellbeing.

In Model 1 and 2, perceived supervisor support and perceived social climate were alternately entered as the dependent variable. The examined covariates were stepwise entered into both regression models. The three socio-demographic control variables were entered into the models in step 1, and the independent study variable (i.e., self-reported talent) in step 2. SPSS version 27 was used to conduct the statistical analyses.

The results presentation includes logit coefficients (*B*) with respective standard errors (SE); the Wald chi-square statistic (Wald); *p* value (*p*) where *p* = <0.05 is reported as statistically significant, and odds ratios (OR) with related 95 % confidence intervals (CI). Cox and Snell's R-Square and Nagelkerke's R-Square are used to report the improvement in model likelihood over the null model.

## Results

In Tables 2, 3, we present the regression results for Model 1 and Model 2, respectively. These results indicate that none of the three socio-demographic control variables (i.e., age, gender, and supervisory position) were statistically significant covariates of social wellbeing (operationalized by perceived supervisor support and social climate) in any step of the models at the *p* < 0.05 significance level.

**Table 2:** Stepwise logistic regression Model 1: Perceived supervisor support regressed on socio-demographic control variables and self-reported talent (*N* = 618).

	***B***	**SE**	**Wald**	**df**	***p***	**OR (CI)**
Step 1						
Age = over 50	0.16	0.25	0.40	1	0.53	1.17 (0.72–1.89)
Age = 41–50	0.29	0.24	1.46	1	0.23	1.34 (0.83–2.15)
Age = 31–40	0.13	0.27	0.22	1	0.64	1.13 (0.67–1.92)
Age = 30 or under				3		
Gender	−0.20	0.18	1.25	1	0.26	0.82 (0.58–1.16)
Supervisory position	0.15	0.20	0.61	1	0.44	1.16 (0.79–1.71)
Constant	−0.68	0.22	9.29	1	0.00	0.51
Cox and Snell *R*^2^			0.005			
Nagelkerke's *R*^2^			0.007			
Step 2						
Age = over 50	0.22	0.25	0.79	1	0.38	1.25 (0.76–2.04)
Age = 41–50	0.36	0.25	2.18	1	0.14	1.44 (0.89–2.33)
Age = 31–40	0.32	0.28	1.32	1	0.25	1.38 (0.80–2.37)
Age = 30 or under				3		
Gender	−0.24	0.18	1.76	1	0.19	0.79 (0.55–1.12)
Supervisory position	−0.04	0.20	0.04	1	0.85	0.96 (0.65–1.43)
Self-reported talent	0.84	0.19	20.58	1	0.00	2.32 (1.61–3.34)
Constant	−1.18	0.26	21.55	1	0.00	0.31
Cox and Snell *R*^2^			0.04			
Nagelkerke's *R*^2^			0.05			

The probability for perceived supervisor support = dependent variable (dichotomized into 1 = high social wellbeing and 0 = low social wellbeing); OR, odds ratio; CI, 95 % confidence interval. The socio-demographic control variables were entered in step 1, and self-reported talent in step 2.

**Table 3:** Stepwise logistic regression Model 2: Perceived social climate regressed on socio-demographic control variables and self-reported talent (*N* = 618).

	***B***	**SE**	**Wald**	**df**	***p***	**OR (CI)**
Step 1						
Age = over 50	−0.38	0.25	2.27	1	0.13	0.68 (0.41–1.12)
Age = 41–50	−0.34	0.25	1.87	1	0.17	0.71 (0.44–1.16)
Age = 31–40	0.09	0.27	0.10	1	0.75	1.09 (0.65–1.84)
Age = 30 or under				3		
Gender	0.25	0.19	1.71	1	0.19	1.28 (0.88–1.86)
Supervisory position	−0.05	0.21	0.06	1	0.81	0.95 (0.63–1.43)
Constant	−0.82	0.23	12.96	1	0.00	0.44
Cox and Snell *R*^2^			0.01			
Nagelkerke's *R*^2^			0.02			
Step 2						
Age = over 50	−0.34	0.26	1.71	1	0.19	0.71 (0.43–1.18)
Age = 41–50	−0.30	0.25	1.36	1	0.24	0.75 (0.45–1.22)
Age = 31–40	0.26	0.27	0.90	1	0.34	1.30 (0.76–2.22)
Age = 30 or under				3		
Gender	0.22	0.19	1.35	1	0.25	1.25 (0.86–1.82)
Supervisory position	−0.23	0.21	1.15	1	0.28	0.80 (0.52–1.21)
Self-reported talent	0.77	0.20	15.61	1	0.00	2.16 (1.48–3.17)
Constant	−1.29	0.26	23.94	1	0.00	0.28
Cox and Snell *R*^2^			0.04			
Nagelkerke's *R*^2^			0.05			

The probability for perceived social climate = dependent variable (dichotomized into 1 = high social wellbeing and 0 = low social wellbeing); OR, odds ratio; CI, 95 % confidence interval. The socio-demographic control variables were entered in step 1, and self-reported talent in step 2.

Both our hypotheses posited that there is a significant association between self-reported talent and employee social wellbeing, showing higher odds of high levels of social wellbeing for self-reported talents than for self-reported non-talents. In step 2 of Model 1, we found support for *H1*, in which social wellbeing was operationalized by perceived supervisor support. Specifically, the odds for self-reported talents were 2.32 times higher than for self-reported non-talents.

Similarly, *H2* was confirmed in step 2 of Model 2, where social wellbeing was operationalized by perceived social climate. The odds for self-reported talents were 2.16 times higher than for self-reported non-talents.

## Discussion

Questions about the value of TM remain understudied in current research. In the study presented here, we focused on a central TM practice (i.e., talent identification) and a key dimension of employee wellbeing (i.e., social wellbeing), as it is frequently proposed to be the most important value of the less studied, inclusive approach. Logistic regression analyses were chosen in correspondence with the study aim, which was to explore the association between self-reported talent and perceived social wellbeing at work. Applying logistic regression analysis methods allowed for exploring the association of interest by comparing the odds of belonging to the first category for respondents who perceived that their organization recognized their individual talents with the odds for those who did not. The study was based on four Finnish companies, all of them representing the manufacturing context and adopting an inclusive TM approach. In both our hypotheses, we assumed that the odds for self-reported talents of belonging to the category in which respondents perceived high levels of social wellbeing were higher compared to those for self-reported non-talents. Social wellbeing was measured in terms of perceived supervisor support in the first model, and in terms of perceived social climate in the work unit in the second model. In the analyses conducted, both hypotheses were supported.

Specifically, the perception that one's individual talents have been identified for long-term deployment by the organization was significantly associated with both social wellbeing variables. Thus, the empirical results of the present study provide tentative support to the proposition that inclusive TM creates value through the identification of employees' individual talents as this practice can be associated with their enhanced wellbeing (Meyers, 2016), at least in terms of their increased social functioning at work.

Further, self-reported talent was slightly more strongly associated with perceived supervisor support than with perceived social climate in the work unit. We can only speculate why this was the case. However, it may be that the organization's long-term investments in and prioritization of talent (which are manifested in practices such as talent identification) are more strongly associated with support from the supervisor since supervisors act as agents of the organization (Rhoades and Eisenberger, 2002) in the implementation of TM in practice. Although the organization can strive to put identified talents into teams and units in which they believe a social climate is created that can promote the talents' functioning, the association between the organization's talent decisions and social climate might be weaker since, ultimately, social climate is co-created by the employee and the people working in the same work unit (Sundin et al., 2006).

Also, it is worth noting that none of the three socio-demographic control variables in the present study were significantly associated at the *p* < 0.05 significance level with any of the included social wellbeing variables.

Next, according to our descriptive results, a relatively high number of respondents reported that their individual talents have been recognized by the organization they work for. The inclusive TM approach is characterized by broad investments in talent across the workforce (Meyers, 2016), and our results can be interpreted to suggest that the implementation has been successful in the studied organizations. To elaborate, it seems that the studied organizations have been sincere in their efforts to identify each employee's individual talents and assess how these are compatible with the long-term needs of the organization (i.e., P-O fit) as well as current and future roles within the organization (i.e., P-J fit). In case an employee has been assessed to fit into the organization (as well as current and future roles), this decision has successfully been communicated. The identification then signals that the organization values the individual talents of the employee and supports the deployment and development of these talents. The experience of being valued and appreciated, in turn, makes the self-reported talents perceive high social wellbeing.

Finally, although we found higher odds of perceived social wellbeing for self-reported talents than for self-reported non-talents, it should be noted that the overall mean for the whole sample (i.e., including both groups) with regard to both the examined social wellbeing variables can be considered relatively high. Thus, self-reported non-talents did not score low on the examined social wellbeing variables either. Our interpretation of this finding is related to the Nordic work context. Similar mean scores have been reported by researchers in other studies examining supervisor support and social climate in the Nordic work context, and which have used the QPS-instruments applied also in our study (e.g., De Cuyper et al., 2011; Jönsson et al., 2015; Sigursteinsdottir et al., 2020). It is therefore possible that the relatively high mean scores presented in this study at least partially are results of health-promoting leadership, which is an important feature of the Nordic welfare system (Eriksson, 2011). Of course, high levels of wellbeing as a general standard among workers should be considered a good thing. However, this can also be considered a challenge for researchers studying the Nordic work context since it becomes more complicated to identify significant groupwise differences.

### Implications

The literature on TM has hitherto discussed positive psychology as well as the literature on P-O and P-J fit as a promising theoretical basis for the inclusive approach (e.g., Swailes et al., 2014) and shown how it stands in stark contrast to extant frameworks rooted in the exclusive approach (Meyers, 2016). To the best of our knowledge, the present study is the first to adopt this theoretical framework and use statistical methods to examine whether a central practice of inclusive TM (i.e., talent identification) delivers on its promises, which above all else is to enhance employee wellbeing (Swailes et al., 2014). As we have shown in this study focusing on the social dimension of employee wellbeing, the perception that one's individual talents are being recognized by the organization for long-term deployment is associated with the perception of high supervisor support and a positive social climate in the work unit. Two main implications of these findings are discussed below.

First, our findings suggest that the “net effects” of talent identification in inclusive TM are high. That is, although self-reported talents benefit from being recognized for their individual talents by the organization, it is not at the expense of self-reported non-talents, thanks to the within-person perspective incorporated in inclusive TM (Meyers, 2016). In addition, the organization benefits from the identification of employees' individual talents in terms of the likely retainment of a workforce with high wellbeing that matches the characteristics and abilities that the organization needs for its long-term success (Allen et al., 2010; Ahl et al., 2018). In comparison, research advocating the exclusive TM approach has been criticized for not presenting convincing evidence that the advantages of normative talent identification outweigh potential disadvantages (Meyers, 2016).

The second implication of our findings concerns the self-reported non-talents. Although the self-reported non-talents' social wellbeing was in no way alarmingly low, they still reported lower levels of social wellbeing compared to the self-reported talents. Thus, organizations adopting an inclusive TM approach should make serious efforts to address the situation of the self-reported non-talents case by case. For some of these employees, the provision of sufficient training and opportunity could unlock their individual talents and enhance their (social) wellbeing. For others, it might be that they would find a better fit in another organization, and their current employer should in such cases promote a successful transition (Swailes et al., 2014). If it also considered the needs of the self-reported non-talents, talent identification in inclusive TM could become even more pluralistic.

### Limitations and future research

The present study is not without limitations and future research is needed. We outline three limitations related to the study data, which were based on employee perceptions.

First, even though we tried to reduce the risk of common method bias, for example by clearly communicating that study participation was voluntary, confidential, and anonymous, our results should be interpreted with caution. Alternative approaches would have been the use of objective measures or the reports of external evaluators. However, this study specifically explored the value of talent identification in inclusive TM from the employee perspective.

Second, although the individual employee was the data source for both the independent and the dependent variables, the former was a grouping variable and the latter based on items scored on Likert scales. Also, the question used to measure the independent variable was placed in the beginning of the questionnaire as a background question. Hopefully, a reasonable degree of psychological separation was created using this questionnaire design, which subsequently reduced other potential sources of common method bias (Podsakoff, 2003).

Third, there might be a discrepancy between how the talent identification was perceived by the employee, how it was intended by the organization's decision-makers, and how it was implemented by yet other agents of the organization. However, following a growing number of TM scholars, we argue that it is the perception of being recognized for one's individual talents that is associated with enhanced social wellbeing and any other potential outcome at the individual level, rather than how this practice was intended or implemented (Thunnissen, 2016). Nevertheless, future studies are encouraged to examine how the employee perception of talent identification (i.e., self-reported talent) is correlated with the intended and actual talent identification implemented by the organization.

Further, in line with common practice, we operationalized employee social wellbeing in terms of social support from the supervisor and social climate in the work unit (Grant et al., 2007), and we used instruments from an internationally validated questionnaire (Dallner et al., 2000) to measure this construct. To further advance the understanding of how self-reported talent is associated with employee social wellbeing, future research should study other sources of social support (e.g., organizational support) or operationalize employee social wellbeing differently (e.g., by co-operation). While the inclusion of additional variables would have been out of scope for the current study, we also encourage studying potential mediators and moderators of the relationship between self-reported talent and social wellbeing (e.g., employees' inherent resources, such as self-efficacy), as well as other dimensions of employee wellbeing (e.g., psychological wellbeing).

In the light of our results, we advocate further use of positive psychology as well as P-O and P-J fit theory as a theoretical framework in empirical examinations of issues related to inclusive TM. We considered the use of a cross-sectional design appropriate for this paper since we are not aware of any prior studies that have studied the association explored in this paper. While this study design allows us to demonstrate that the examined variables are associated with each other (and thus take an initial step in the examination of whether and how these variables are interrelated), it does not allow us to make causal inferences.

Specifically, it might be that employees who reported high social wellbeing in this study are more likely than other employees to be identified as talents by the organization, rather than the other way around. In this interpretation, the employees may have embraced existing opportunities and ensured that they benefit from supportive work circumstances in a way that has made their individual talents visible to the organization. However, no matter how well the employees have succeeded in making their individual talents visible to the organization, this will likely have little impact on the organization's decision-making about talent identification if the talents of an individual don't fit into the organization. To some extent, it is thus feasible to argue that reverse causality resides in employee proactivity.

Nevertheless, our finding that the examined variables are associated with each other justifies the extensive time and effort that will have to be invested in future longitudinal research (which we advocate) to clarify the direction of the relationship between them.

Further, the findings of this study should be interpreted bearing in mind that the study results were based on a Finnish sample. The Nordic welfare model has clearly contributed to Finland's longstanding working life traditions of inclusive, egalitarian, and participative management (Ahl et al., 2018), and this might explain why an inclusive TM approach seems to be well-received among the employees of the companies in this study. The working population that this study aimed to target, i.e., employees in the manufacturing industry, should also be kept in mind when interpreting the results. Although we used a representative sample of the participating organizations' total populations, for example with regards to the distribution of age, gender, and supervisory position, the results of the study may not be fully applicable to other country, industry, and sector settings. Hence, future studies should replicate our findings in other settings.

Finally, the COVID-19 pandemic inevitably has impacted the labor market. For example, remote work has become more common, changing the way individuals interact with each other at work. This change should be considered when assessing the implications of the study findings. However, it is our belief that the perception of being recognized for one's individual talents is likely to remain associated with increased social wellbeing even when individuals work remotely since individual talents can be recognized and social connections can be established, developed, and maintained with the help of technological tools although individuals are not physically located at the same place. For example, in remote work encouraging feedback may be communicated through email rather than face-to-face, but regardless of how the feedback is communicated it is likely to indicate social support.

## Conclusion

In conclusion, our results evidenced an association between employees' perception of being recognized for their individual talents by the organization and their perceived social wellbeing at work. Based on our findings, we tentatively support the proposition that inclusive TM creates value through the identification of employees' individual talents as this practice can be associated with their enhanced wellbeing, at least in terms of their social wellbeing at work. Also, we advocate the use of positive psychology as well as P-O and P-J fit theory as a theoretical framework for empirical examination of issues related to inclusive TM practices.

## Data availability statement

The datasets presented in this article are not readily available because they are part of a larger research project conducted in 2018. The datasets will be made available upon request. Requests to access the datasets should be directed to JB, janina.bjork@abo.fi.

## Ethics statement

Ethical review and approval was not required for the study on human participants in accordance with the local legislation and institutional requirements. Written informed consent for participation was not required for this study in accordance with the national legislation and the institutional requirements.

## Author contributions

JB formulated the aim of the study and the related hypotheses, designed the survey, collected as well as applied statistical techniques to analyze the study data, communicated with participating organizations, prepared the published work, wrote the initial draft, revised as well as edited the manuscript, and acquired the financial support for the publication of this work. PB participated in the discussions around survey design, in addition to contributing to the preparation of the published work, specifically with critical reviews and revisions of the various versions of the manuscript. AF participated in the discussions around survey design and data collection, applied statistical techniques to analyze the study data, and contributed to the preparations of the published work, specifically with critical reviews and revisions of the various versions of the manuscript. All authors contributed to the article and approved the final, submitted version.

## Funding

This research work was funded by the Finnish Work Environment Fund (Grant Number: 200264) and Svensk-Österbottniska Samfundet (a regional research and development funding instrument).

## Conflict of interest

The authors declare that the research was conducted in the absence of any commercial or financial relationships that could be construed as a potential conflict of interest.

## Publisher's note

All claims expressed in this article are solely those of the authors and do not necessarily represent those of their affiliated organizations, or those of the publisher, the editors and the reviewers. Any product that may be evaluated in this article, or claim that may be made by its manufacturer, is not guaranteed or endorsed by the publisher.
